# Barriers and facilitators of integrated hepatitis B, C, and HIV screening among pregnant mothers and their newborns attending maternal and newborn clinics in Koboko District, Uganda: A qualitative inquiry of providers’ perspective

**DOI:** 10.21203/rs.3.rs-3739602/v1

**Published:** 2023-12-18

**Authors:** John Bosco Alege, John Paul Oyore, Rose Clarke Nanyonga, Philippa Musoke, Alloys S.S Orago

**Affiliations:** Kenyatta University; Kenyatta University; Clarke International University; Makerere University Kampala; Kenyatta University

**Keywords:** Barriers, facilitators, Integrated, screening, maternal and newborns

## Abstract

**Background:**

HIV and HBV remain significant public health challenges characterized by high prevalence, morbidity, and mortality, especially among women of reproductive age in Uganda. However, both HIV and HBV patients are managed in separate clinics with separate staff even though they all receive ART. Patients with HBV do not receive routine counselling and education, and there are limited resources for laboratory investigation coupled with a high loss to follow-up. This study set out to “assess barriers and facilitators of integrated viral hepatitis B C and HIV care model to optimize screening uptake among mothers and newborns at health facilities in Koboko District, west Nile sub-region, Uganda”.

**Methods:**

A cross-sectional grounded theory qualitative approach was employed in an institutional setting (HC IIIs). Data was audio recorded using a recording device during the key informant interviews and was transcribed after all interviews were conducted. Data was then analyzed using framework analysis.

**Results:**

The following facilitated integration: High prevalence, and therefore burden of hepatitis B infection in West Nile region, team spirit by the health workers, reduced long waiting time, availability of medical products such as HBV and HCV test kits, integration of HBV and HIV into HMIS2 form and availability of support from implementing partners such as Infectious Dease Institute which offered mentorship and training on integration and support supervision.

**Conclusion:**

Barriers to integration included; knowledge gap among health care workers, lack of transport for patients, language barriers during health education, inadequate human resources for health, stock-out of testing kits for HBV and HCV, lack of HMIS 2 column to capture HCV data, lack of funds to facilitate follow up of patients after referral for further investigation upon suspected cases of HBV and HCV. The study participants recommended; Promoting the integration of HBV, HCV, and HIV into routine health services; ensuring a constant supply of HBV, and HCV test kits to avoid stock-out; Engaging VHTs/Community health volunteers to support follow-up of patients and conducting health care workers performance reviews; addressing the issue of inadequate human resource; and finally dealing with misconceptions at community level about HBV and HCV diseases which hinder access to services.

## 1. Introduction

Hepatitis B virus (HBV) and HIV are the leading cause of chronic viral infection globally and it’s among the infections with prominence in the clinical evaluation of pregnancy [1]. HBV infection in pregnancy poses serious implications such as the risk of development of chronic HBV, perinatal transmission of HBV and accelerated HIV-related liver damage [2].

Globally, there were about 2 billion people infected with HBV one-third of whom reside in China [3]. China is also one of the countries with a higher prevalence of Hepatitis C virus (HCV) infection with an estimated burden of 9.8 million infections [5]. According to the Joint United Nations programs on HIV/AIDS (UNAIDS) and World Health organization (WHO), about 38 million people were diagnosed with HIV, 257 million had HBV and 71 million had HCV by the end of 2020 [6]. In addition, the WHO and UNAIDS implemented strategies to promote the global elimination of these viruses by 2030 [7]. The success of the strategies relies on testing and diagnosis of at least 90% of all persons living with HBV, HCV and HIV infection, and steps towards engagement in care and treatment [8]. In Nepal the burden of HBV/HIV, HIV/HCV, and HIV/HBV/HCV remain very high at a rate of 2.995%,18.14%, and 2.53% respectively [5].

The African continent is currently facing a double burden of HBV and HIV which has affected approximately 5 to 8% of the continent’s population [9]. In addition to HIV, HBV remains a significant public health issue characterized by high prevalence, morbidity, and mortality [10]. Moreso, Africa accounts for 26% of the global burden of hepatitis B and C and 125,000 associated deaths [11]. Similarly in Africa, the coverage for routine childhood vaccination against hepatitis was 72% well below the target of 90% required to ensure that the virus is no longer a public health menace. In addition, HBV birth dose is administered in only 14 countries in Africa with an overall coverage of 10%. Relatedly, about 2% of persons infected with hepatitis B were diagnosed while only 0.1% were treated and 5% of persons infected with HCV were diagnosed while close to 0% were treated in Africa[11].

In Kenya, the prevalence of HBV co-infection with HIV was 5.8% (95%CI:2.65 to 8.9%) while HCV co-infection was 4.2% (95%CI:1.65 to 7.4%)[12].

In Uganda, there is a regional variation in the prevalence of both HIV and HBV infections. In Northern Uganda, the prevalence of HIV was 3.1–7.2% and the one of HBV was 6–12%. in contrast, there was lower HBV prevalence in Southern Uganda ranging from 4.7–8%[13–14]. Despite the high prevalence of HBV and HIV in the country, a study conducted on the uptake of HBV vaccines at birth established (39.68%) in north Uganda compared to 0.23% in the central and eastern parts of Uganda %)[15].

Uganda like any other sub-Saharan African country has a functional infrastructure and service delivery system for HIV care except for HBV [16]. As of now, both HIV and HBV patients are managed in separate clinics with separate staff teams even though they all receive antiretroviral treatment. Patients with HBV do not receive routine counselling and education, there are limited resources for laboratory investigation, and a high loss to follow up. HBV-infected persons are not being tested for HIV and they do not undergo routine hepatitis testing or education, yet HIV services have been integrated into routine services at the primary care level in most African countries [16].

The key component to reducing morbidity and mortality among people living with HBV is to receive early diagnosis and clinical care. In addition, integrated care models include follow-up of patients, knowledge on triple elimination of HBV, HCV and HIV, diagnosis of HBV, HCV and HIV, the effectiveness of diagnostic methods, vaccination, the effectiveness of treatment and prevention of HBV, HCV, and HIV [17].

Against this background, the study sought to assess barriers and facilitators of integrated viral hepatitis B C and HIV care model to optimize screening uptake among mothers and newborns at health facilities in Koboko district, Uganda.

## 2. Methods

### Study setting

A cross-sectional grounded theory qualitative approach was employed in an institutional setting to obtain data on facilitators and barriers of integrated viral hepatitis B, C and HIV screening among pregnant mothers and their newborns in selected health facilities in Koboko, district, West Nile sub-region. Data was audio recorded using a recording device during the key informant interviews and was transcribed after all interviews were conducted. This type of approach was used because it provides explicit, chronological guidelines for conducting qualitative research, offers specific strategies for handling the analytical phases of inquiry, streamlines and integrates data collection and analysis and advances conceptual analysis of qualitative data [18].

### Study design and sample selection

This study was conducted at the level of HCIIIs and the general district hospital. In Uganda, a health center III is managed by a Clinical Officer, it serves a population of 10,000 people and acts as a referral for Health Centre IIs as well as offering in-patient care, and simple diagnostic and maternal health services [19]. The study population comprised of participants drawn from; health facilities in-charges, research assistants (midwives/nurses), DHO, district EPI focal person, ART coordinator, district medical laboratory coordinator, and district cold chain technician.

### Diagnostics

The HBV and HCV testing kits were supplied for testing pregnant women in their first trimester. The HIV testing kits (DUO), coupled with the vaccines were already in the study sites supplied by MoH.

Respondents for this study were selected purposively based on their involvement in the delivery of HBV, HCV, and HIV care services to both neonates and mothers. Qualitative samples are purposive by virtue of the respondents having the capacity to provide rich-textured information that is relevant to integrated health services delivery [20]. The sample size determination was guided by the criterion of informational redundancy, when no information is generated by sampling more units, the sampling of respondents can be terminated. This follows the logic of informational comprehensiveness, which that suggests the more information power the sample provides, the smaller the sample size is required during the study [21]. Therefore, 20 key informants participated in this study. A researcher-administered technique through face-to-face interaction was used for collecting data during the key informant interviews (KIIs).

### Study tools and data collection

The KII was used for collecting data from the study participants. The triple elimination for HBV, HCV, and HIV was measured using the key indicators.

### Data analysis

The data was audio recorded using phones during the KIIs, then it was transcribed after all the interviews were conducted. The data was then analyzed using framework analysis as it allows systematic data review and allows researchers to ensure that they handle the data per predetermined procedures [22]. The coding process was done using an inductive method that generates emerging themes while a deductive approach was used for pre-selected themes.

The study protocol was reviewed, and approved by Clarke International University Research Ethics Committee, as part of a bigger study on integrating HBV, HCV and HIV, and then registered with Uganda National Council for Science and Technology under registration numbers: CLARKE-2022–388, and HS2706ES respectively.

Permission to access the study participants and the health facilities was granted by the Chief Administrative Officer of Koboko district. Written voluntary informed consent was obtained from each respondent and the purpose of the study was. Participation in the study was voluntary and one could withdraw from the interview at any stage. Confidentiality of data collected was ensured to protect the privacy of the study participants. Utmost respect was accorded to the study participants.

## 3. Results

A total of (20 KIs) participated in this study; (12) were male, while (8) were female. Similarly, (10) respondents were aged 25 to 30 years while the remaining (10) were aged 31 to 45 years and above. In terms of the cadre (2) of the study participants were enrolled midwives, 8 were clinical officers, (1) was a district laboratory focal person, and (1) was the ART Focal Person at Koboko District General Hospital 8 were nurses (enrolled and registered). In terms of their positions at the time of the interview, (5) were maternity in-charges, (5) facility in-charges, (1) was a District Laboratory Focal person, another (1) ART/Hepatitis Focal Person at Koboko district general hospital, and (1) was the District Cold chain focal person.

Facilitators and Barriers of integrated viral hepatitis B, C and HIV screening among mothers and newborns

The facilitators and Barriers of integrated HBV, HCV, and HIV at one point of care were assessed using the WHO Health Systems Framework based on the six building blocks of a healthcare system that include; service delivery, health workforce, health management information system, medical products vaccines and technology, financing, and leadership & governance. Several sub-themes emerged as facilitators and barriers to integrated HBV, HCV, and HIV as discussed under each study objective and building block below.

- Facilitators of integrated viral hepatitis B, C, and HIV screening among mothers and newborns

### Service delivery

#### Awareness and general knowledge about integrated health services delivery

Overall, the study participants were aware of the integrated health service delivery, and the common themes that emerged were offering integrated and a variety of health services in the health facilities. Integration also involves bringing different services together to achieve one goal, and a means of offering various packages of health services to a particular patient, and or merging health services together.

A respondent was quoted saying:

*The initial plan can be HBV but we bring HIV, syphilis, HB estimation (check blood level), check syphilis, and blood grouping. Knowing the HIV status of the mother is important so as to protect the child and have an early intervention.* Another respondent also stated *“It means offering various packages of health services to a particular patient for example” physical examination of mothers, screening for HIV, HBV, malaria, syphilis, HCV, offer appropriate treatment to those found diseased.* In addition, *“we also give them health education, talk to them about the importance of conducting screening at the maternity and out-patient department (KI).*

This study established that the majority of the respondents had ever heard about integrated services for viral diseases, and they acknowledged offering services to pregnant mothers, especially for HIV and HBV services. Services provided include; screening of mothers at ANC and maternity for; HIV, and HBV, linking HIV positive clients to care, and referral of HBV patients for further investigation to Koboko General Hospital. One respondent noted that

For the three viral infections, we screen for clients who come for health care services depending on the case history/assessment for, HBV and HIV. We do confirmatory test in the OPD laboratory for clients who test HIV positive and link them to care in the ART clinic. However, under public service delivery system, we do not have test kits for HCV” we have only accessed them through this project” (KI).

On the contrary, one participant disagreed that the integration of the three viral diseases (HBV, HCV, and HIV) is less talked about to the patients. The respondent was quoted saying *“At my facility, HBV is one of the conditions that are less talked about and we normally suspect HBV when there is yellowing of the eyes, abdominal distention”. “We do not have a clinic for HBV, Our ART clinic only incorporates TB and leprosy in the ART clinic (KI).*

#### Diagnostics (screening), health education, and linkage to care

The severity of hepatitis B infection in the region and serving humanity to protect the mother and the newborn are some of the reasons why health workers promote integrated HBV, HCV, and HIV. It was also reported that HBV services are integrated into routine immunization services mainly for newborns.

“Involvement of implementing partners such as Infectious Diseases Institute (IDI) which offered onsite mentorship and support supervision of the health care workers has increased the number of patients seeking HBV services (KI).

The study participants further elaborated that for clients who test HBV (+) positive, they always subject them to other preliminary tests like antigen tests, and abdominal scans to establish whether there is an impact on the liver. One respondent said; *Depending on the outcome, we automatically start them on either medication or lifestyle counselling”. However, “under the current study, we only screen pregnant mothers during their first ANC and first trimester, if we find them positive, we refer them to the general district hospital for further investigation to reaffirm the positivity (KI).*

Another respondent stated that *“In a nutshell, our West Nile region is where viral hepatitis is still a problem as a region and we feel we have a burden that we should manage. In addition, “Integration has become policy of Ministry of Health and it has to become part of us, as we offer services to the clients.*

Integration has built our capacity as health care workers, we used to think HBV testing was meant for laboratory personnel but through integration, the midwives can now do HBV testing (improved level of knowledge for midwives)” and it has given us opportunity for early detection of viral diseases.

#### Linkage to care

The following were established on linkage to care for those who test positive for HBV and HIV;

*We do confirmatory test and link them to care and they are initiated on ART* For HBV-positive clients, *we refer them for confirmatory test to the district general hospital for example, the ART clinic at the hospital conducts antigen. We do viral load twice a year to determine replication of the virus (KI).*

While for exposed infants to HIV and HBV, nevirapine syrup is given to them to prevent mother-to-child transmission. *Children exposed to high-risk mothers are initiated on nevirapine syrup and PCR test are done at 6 and 8 months to establish whether the child acquired the virus and if PCR results turn negative, we discontinue the syrup”* Meanwhile, *for children exposed to HBV positive mothers, we do not vaccinate the baby at birth but we establish whether the baby has acquired the virus, we get samples and send them to the district general hospital for further investigation (KI).*

### Health workforce

This study established that team spirit from health care workers facilitated integrated services for viral diseases care. This is because fellow health care workers “cover up” to fill in the gap for their colleagues in case, they are taken by other duties outside the health facility. Additionally, personal commitment from health care workers’ perspective and involvement of implementing partners through training of health care workers, supervision and supply of testing kits enhanced integrated health services delivery. To affirm that statement, one participant elaborated this further

*Different stakeholders and implementing partners supported health care workers for the good work done in various ways; for example, some patients gave a vote of thanks to the staff, while implementing partners provided capacity building, on-site mentorship during integrated out reaches, provision of ‘safari day’ allowance, transport allowance and salary increments by the ministry of health Uganda, that positively influenced health care workers attitude towards integrated viral hepatitis B, C and HIV (KI).* On team spirit one respondent stated that

When the ANC department is overloaded with work, the general laboratory personnel always come in to support especially in testing”. Also, “External quality assurance checks on the competence of health care workers to establish whether the reagents are working very well, we then give feedback which makes health care workers very happy” (KI)

### Medical products, supplies and vaccines.

The majority of the KIs acknowledged that medical products such as testing kits for HBV and HIV were available in most of the health facilities and were accessible to every patient. However, no HCV RDTs are currently supplied by MoH. A respondent stated

We get supplies from national medical stores every two months; the consignment is first taken to the district but each facility has its package labeled (KI).

### Health management information system (HMIS)

This study established that at source document and point of care level data is managed by the respective departments such as; Outpatient (OPD), laboratory, ANC and maternity. The responsible persons have been assigned to handle data in the respective departments. It was noted that; *There are no challenges at all, the test results delivery depends on turnaround time for liver function and renal function tests, we normally collect enough samples (blood) for testing from the patients and run tests at the same time like in the following day (KI).*

### Leadership and governance

Regarding the leadership and governance building block; the health facility In-charges have the mandate to make requisitions for test kits, and vaccines on behalf of their respective lower health facilities such as the Health Centre IIIs in case of shortages. While the District Health Office makes requisitions on behalf of all the health facilities including Koboko General Hospital. *We make requisition, sometimes we borrow from other nearby facilities when there is a shortage, and also the implementing partner Infectious Disease Institute (IDI) usually help us with some of the supplies (KI).*

- Barriers to integrated viral hepatitis B C and HIV care among mothers and newborns.

### Service delivery (health education, screening, linkage to care)

#### Health education

There are several sub-themes that emerged as barriers to integrated service delivery. These included; a shortage of testing kits, lack of Information Education materials (IEC), language barriers, limited knowledge on HBV/C, and inadequate staffing. While barriers to linkage to care included; a lack of funds for follow-up of patients, transport challenges to support referral of patients to the next level, and inadequate knowledge among the village health teams at the grassroots level on integrated HBC, HCV, and HIV. It was noted that

When it comes to the issue of testing, the midwife feels it should be done by laboratory personnel and the laboratory personnel says, since the client is pregnant, she should be tested by the midwife and eventually leading to long waiting time from both the maternity and main laboratory (KI)

In addition, it was revealed that *“For HCV; we have knowledge gap because we are used to doing integrated testing for HIV and HBV and when HCV was introduced, in this project we had a gap and we took some time without doing the test” While another respondent noted that “Health care providers lack information on HBV/ C, they do not offer sufficient information to the clients.* Consequently, they (midwives and nurses) are not willing to test pregnant mothers regularly since health talks are organised on different topics and offering targeted health talk is not sufficient at integrated care points. Thus, a respondent stated that *“There is inadequate knowledge among the village health teams on HBV, HCV and HIV during health visit to the pregnant mothers and the community (KI).*

### Shortage of test kits and vaccines

#### Shortage of test kits;

Shortage of test kits emerged as a significant barrier to the integration of viral diseases. This is because the government stopped supplying them in 2018. It was during this study project that we got some HBV/HCV test kits but are only meant for pregnant mothers in their first trimester. One respondent stated *“government does not supply testing kits for HCV but HBV is supplied occasionally and the available testing kits only target pregnant mothers yet there are other people who need the services (KI).*

#### Language barriers;

Relatedly, the study established that language barrier is a real challenge during health education. This is because patients in Koboko district (study area) speak various languages such as; Lugbara, Kakwa, Lingala, and Arabic which the healthcare workers cannot speak fluently during health education sessions. As a result, when there is no interpreter, health education sessions are not conducted, and health care workers start offering services without appropriate or adequate information.

We have VHTs who help us interpret but the days VHTs are not there, we start providing services without health education because there is no one to interpret. We also lack IEC materials in local language such as in South Sudanese and Congolese commonly spoken languages (KI).

#### Diagnosis/screening and linkage to care

Linkage to care mostly at the referral level was found to be a barrier, and this is evidenced in the following narrative *Patients take long to come to the general hospital upon referral from the lower-level facility due to high transport cost. Initially we had challenges linking clients from maternity to OPD. For some patients we had to escort them to the point of care (KI).*

### Human resources for health

#### Inadequate staffing

Human resources for health emerged as a significant barrier to the integration of viral hepatitis B, C, and HIV services in all the health Centre’s III in Koboko district. The respondents experienced inadequate staffing at different points of care mostly during community outreaches, at maternity, OPD and during health education. For instance, one respondent had this to say; *We have limited number of healthcare workers, for maternity, we have two volunteers who do not come every day and when they come, they spend 20–30 minutes, and they leave the health facility because they are not paid salary. (KI)*

### Health management information system

Generally, all respondents had issues with the health management information system because the HMIS 2 does not have a provision/column for entering information on HCV and as a result, HCV records are captured in counter books and sometimes papers improvised by the health care workers. Moreover, some details of the patients are taken at the point of care for example, place of residence, and the ANC preliminary care makes follow up very difficult. Also, HBV and HCV are not integrated into the Electronic Medical Records which makes updating records in the system very difficult.

### Leadership and governance

At the leadership and governance level, respondents experienced several barriers including delays in re-stocking of HBV adult vaccines. Rampant transfer of health workers providers already trained in the integration of the three viral diseases as well as political influence and objection of referrals, loss to follow up, and negative attitude towards external quality assurance by health care providers. One respondent stated that *“Limited human resource causes complaints due to long waiting time, when I am away, any services offered by me are put on hold. Similarly, the hospital management committee is chosen based on technical know-how and some have limited knowledge and understanding making it difficult to work with them (KI).* Administratively, health facilities operate on political boundaries, some areas are outside their catchment area, thus communities seek services from nearby facilities. Integration of skills through internal and external approaches is difficult to manage due to limited resources.

### Recommendations by the Health workers

#### Promote Integration of HBV, HCV, and HIV;

All HBV, HCV HIV, must be integrated into routine services since hepatitis has non-specific symptoms. It was also suggested that implementing partners must promote integration and provide funding for all three viral diseases (HBV, HCV, and HIV). Similarly, there is a need for the office of the DHO to provide transport to facilitate timely referrals. It was also suggested that pregnant mothers must be prioritized at the point of care to avoid delays in accessing services by the duo (mother and baby).

#### Constant supply of HBV, and HCV test kits;

The study participants recommended sufficient and constant supply of HBV and HCV test kits. Similarly, it was suggested that; pregnant mothers and those on first ANC must be tested for HCV; organize outreach programs to offer sensitization for HBV and HCV to create demand for the services, and an additional token or incentives must be given to health workers who handle testing of mothers for HIV, HBV and HCV. It was also suggested that the national medical store should adhere to order cycles and deliver according to schedule.

#### Engaging VHTs/Community health volunteers:

The study participants suggested that VHTs be attached to villages or parishes to support follow-up of patients and conduct health care workers performance reviews. This is meant to establish performance of individual health workers to enhance improvement.

#### Human resources:

It was generally agreed that there is a need to address the issue of human resource. *If we have sufficient number of midwives on ground, we shall offer all required services to pregnant mothers.*

Additionally, training of health workers through on-the-job mentorship and assigning them responsibilities such as providing reports on integration of viral diseases is key. Finally, the involvement of sub-county staff who are key in mobilization during training and mentorship will increase uptake of screening and other HBV, and HCV services.

#### Dealing with misconceptions at community level;

Community dialogues to remove the misconceptions and myths, such as “the baby might not grow well if you go to the health facility at early stages of the pregnancy”. More engagement of key stakeholders such the DHO, CAO, RDC political and religious leaders to support sensitization will increase uptake.

## 4. Discussion

### Facilitators of integrated HBC, HCV and HIV in health facilities

The West Nile region in Uganda has a high burden of HBV and HIV [13]. This study revealed that health workers were aware of integrated health service delivery but reported low knowledge levels on the one-point-of-care approach. They were able to explain aspects of integrated health services by citing several examples including the HBV, HIV and Syphilis combo testing of mothers at ANC. Similarly, participants in this study noted that the severity of hepatitis B infection in the region; the protection of the mother and the newborn from HBV, HCV and HIV infection as some of the reasons that motivated them to integrate screening for the three diseases. Other reasons are the team spirit exhibited among health care workers, involvement of implementing partners in training of health care workers, and support supervision, facilitated integrated services for viral diseases. In a study conducted in an Indian hospital it was found out that health workers were aware of the hepatitis B, and C infection through blood and blood products as a mode of transmission, but awareness in relation to other modes of transmission, and integrated service delivery was dissatisfactory [23]. The HBV and HCV disease burden, variation in the level of healthcare cadre, levels of healthcare facility, differences in testing and treatment protocols may account for the similarities and differences. On the contrary, a study conducted in US identified a knowledge gap on HBV among healthcare providers. The healthcare workers were not aware of the outcome of HBV infection, who should be screened and vaccinated against HBV, the appropriate screening methods as well as interpretation of serologic tests for HBV and proper treatment for the HBV infected [24]. This difference is possible because HBV prevalence in the US is only 0.4% [25]. this is below the WHO endemicity threshold and therefore integration may not be a priority [26].

The availability of HBV and HCV testing kits supplied by this project targeting pregnant women in the first trimester and HIV testing kits (DUO), coupled with the vaccines supplied by the NMS, facilitated the integration of viral diseases service delivery to the pregnant women and newborns. This is consistent with the MoH guidelines were HBV Prevention (vaccination of mothers not infected with HBV given at 0, 1 and 6 months is recommended as well as the introduction of the HBV birth dose [27]. Khan and Rose reported in a study conducted in South Africa that the most cost-effective way to prevent and control hepatitis B is through exposure to vaccine and the perception that the vaccine is safe and effective and can protect for a lifetime [28]. Similarly, a study conducted in the informal settlement of Kampala indicated that having the belief that the hepatitis B vaccine is effective in the prevention of HBV infection influenced uptake of HBV vaccines [29]. Therefore, availability of the medical supplies, the willingness of the healthcare workers to screen, vaccinate and refer patients for treatment, coupled with the positive perception of pregnant mothers promoted integration of HBC, HCV and HIV in the health facilities.

The HBV, HCV, and HIV cascade of care involves several steps such as; screening, diagnosis, linkage to care, assessment of liver disease stage and treatment eligibility. Laboratory blood tests are required at every step of the care cascade, to monitor the health outcomes of the patient [30]. This study established that clients who test HBV (+) positive, are always referred to the general district hospital so other preliminary tests like antigen tests, and abdominal scans can be carried out to establish whether there is an impact on the liver. [31] A study conducted in Australia established that PoC testing theoretically removes several access barriers to services including referrals for continuum of care. Whereas there is available data and evidence for PMTCT for HIV, there is no sufficient data about HBV and HCV especially in the West Nile region and Uganda in general.

In this study, participants reported that availability of the HMIS2 registers and forms, electronic data management information system (EDMIS), dedicated personnel responsible for routine patient data entry into the system by both using the physical HMIS2 form or electronically, contribute to enhanced integrated health services delivery. [32] conducted a study on PMTCT in six provinces in Indonesia, and established that the majority of the districts had insufficient resources to implement PMTCT in the health facilities, including data collection infrastructure. For instance, Health facilities which provided PMTCT were limited and uncoordinated; screening mothers for HIV, HBV and Syphilis was done in Puskesmas (public health centre), while test results that required confirmation as well as study participants who required enrollment on treatment were referred to hospitals or HIV clinics elsewhere. Pregnant women had to come back to Puskesmas for post counselling. More importantly it was difficult to capture, store and transmit data for decision making on integrated services.

In a country with a high prevalence of HBV and lacking nationwide services to prevent mother-to-child transmission, such as Uganda, it becomes imperative to explore innovative approaches, including the integration of HBV and HCV screening and prevention into antenatal care and maternity services. Moreover, gaps exist in availability of literature and evidence on the barriers and facilitators of integrated HBV and HCV in health facilities [33]. In terms of leadership roles by the health facility and maternity In-charges, this study established that requisitions for drugs, test kits, and other medical supplies, and products are made by the health facility In-charges from the district medical store. This eases the bureaucracy and any delays that may be associated with stock-outs, thus enhancing integration

### Barriers to integrated HBC, HCV and HIV in health facilities

This study revealed that a lack of information and education materials (IEC) regarding integrated HBV, HCV, and HIV, along with language barriers stemming from the diverse languages spoken by patients in Koboko district, such as Kakwa, Arabic, Swahili, Lingala, and Lugbara, poses a significant challenge for most healthcare workers. Consequently, conducting effective health education sessions becomes problematic in the absence of an interpreter. Consequently, healthcare workers often provide health services without adequate health education. This, in turn, results in communication gaps during both pre- and post-test counseling, affecting clients who receive positive test results until they enter the continuum of care. A scoping review conducted by Mohanty et al., [34] also established that poor communication between health care providers and caregivers, limited availability and access to health facility-based immunization and cost of vaccines were barriers to integration of viral HBV, HCV and HIV.

Our results demonstrated that healthcare workers exhibited an insufficient level of knowledge regarding the management and treatment of viral diseases, particularly HBV and HCV. This knowledge gap was also evident in their ability to conduct HBV and HCV tests, as they lacked access to comprehensive information and had not received adequate training in viral diseases. Consequently, this deficiency in knowledge and skills hindered the incorporation of HCV screening into pregnancy-related care. [35]. Whereas pregnant women are screened for HBV and HCV, they are not provided with health education particularly at the ANC and maternity. But these challenges seem not to be only an issue in developing countries such as Uganda; for instance, a study conducted in the US identified knowledge gap on HBV among health workers, and reported that they were not aware of HBV infection outcome, who should be screened and vaccinated against HBV, the screening algorithm as well as interpretation of serologic tests for HBV and appropriate treatment for the HBV infected [24]. Additionally, lack of public awareness significantly contributed to poor outcomes from infection among risk persons due to continued transmission to susceptible individuals [36]. Therefore, lack of knowledge was a strong barrier to testing, prevention, and care.

Shortage of test kits for HBV in the health facilities significantly affected integration of viral disease care. Lower health facilities normally have a challenge of capacity to order and store medical products, vaccines and other supplies. A qualitative study conducted in Democratic Republic of Congo established that untimely vaccinations, regular logistics challenges mostly stock-outs, and inability to store vaccines were key barriers to integrated HBV, HCV and HIV screening [37]. Moreso, there were complex and unsynchronized vaccines fees across health facilities, inadequate communication across delivery and vaccination wards, limited and incorrect understanding of vaccines among mothers and community members. A related study conducted in Lira district northern Uganda found that lack of access to HBV screening services at the government health facilities contributed to HBV infection [38]. Medical products, vaccines and technology are a key building block of a healthcare system according to WHO especially in creating access to critical services such as HBV and HCV for mothers.

Shortage of human resources for health to provide HBV and HVC services emerged as a barrier to integration of viral disease care in all the study sites. All study participants highlighted this as a persistent problem both in the maternity and out-patient department. The shortage of human resources for health is a global issue that affects delivery of quality healthcare services. Findings from this study are consistent with those from a study conducted in Indonesia, where researchers established that the majority of the health facilities had insufficient human and other resources to implement PMTCT [38]. In addition, health facilities that offered PMTCT services were limited and uncoordinated. For example, screening mothers for HIV, HBV and syphilis was done in public health facilities while the test results confirmation and treatment initiation were done in other HIV clinics [41] This implies that pregnant mothers had lost opportunities in accessing screening services, treatment and care due to long distance to health facilities, and parallel testing systems due to the insufficient human resources.

This study also established that respondents faced leadership and governance challenges during integration of viral disease cares. Delays were experienced in re-stocking of HBV vaccines for adults, frequent transfer of already trained health care workers in the integration of viral diseases and political influence on decisions to screen every patient for viral disease even when testing kits are meant for pregnant mothers and newborns. Resource mobilization and allocation to facilitate delivery of healthcare services is a function of leadership and governance, the first building block of a healthcare system. A study conducted to assess recommendations for prevention and control of hepatitis B and C revealed that inadequate resources allocated for the prevention, control and surveillance program increased continued transmission of HBV and HCV [24]. Similarly, Ajuwon et al (X) reported in a study conducted in Nigeria that nine in every ten of its population that live with chronic HBV are not aware of their infection status and are not captured in the global health statistics due to lack of resources and lack of political will to address the HBV burden [39].

Finally, loss to follow-up was identified as another challenge in the continuum of care for HBV, HCV and HIV integration. The study participants experienced loss to follow-up of patients due to lack of funds, but also partly due to negative attitude towards clients by the health workers, which negatively affected integration of viral disease care. In a study carried out by Mitchell *et al*., it was established that inadequate resources allocated affected referral mechanism and follow-up of clients [24]. Generally, the majority of health programmes funded by partners tend to focus mainly on procurement of test kits, and renumerations for health workers. However, there is no due consideration to support referral pathways as an important component of promoting the continuum of care.

## Conclusion

The facilitators to integrated viral diseases (HBV, HCV, and HIV) were; high prevalence of hepatitis B infection in West Nile region, team spirit from health workers perspective, reduced long waiting time, availability of medical products such as HBV and HCV test kits, integration of HBV and HIV into HMIS2 form and availability of support from implementing partners such as IDI which offer mentorship and training on integration and support supervision. Barriers to integration included; knowledge gap from health care workers perspective, and lack of transport, language barriers during health education, inadequate human resource for health, stock-out of testing kits for HBV and HCV, lack of HMIS 2 that integrated HCV, lack of fund to facilitate follow up of patients after referral further investigation upon suspected cases of HBV and HCV.

## Data Availability

The raw data for this study are available and shall be shared upon request.

## Abbreviations

ANC: Antenatal Clinic
CHB: Chronic hepatitis B
GHSS: Global health sector strategy
HBsAg: Hepatitis B surface antigen
HBV: Hepatitis B virus
HCC: Hepatocellular carcinoma
MoH: Ministry of Health
REC: Research and Ethics Committee
UNCST: Uganda Council for Science and Technology
